# Case Report: Rare collision tumors: ACTH-secreting pituitary neuroendocrine tumor and pituicytoma – histopathological and ultrastructural analysis

**DOI:** 10.3389/fendo.2026.1793284

**Published:** 2026-04-02

**Authors:** Silvia Carolina Fernández, María Celina Bernhardt, Ezequiel Grondona, Carolina Leimgruber, Virginia Juárez, Ana Clara Venier, María Lorena Bertolino, Emilio Mezzano, Jorge Humberto Mukdsi, Favio Nicolás Pesaola, Ana Lucía De Paul

**Affiliations:** 1 Centro de Microscopía Electrónica, Facultad de Ciencias Médicas, Universidad Nacional de Córdoba, Córdoba, Argentina; 2 Servicio de Patología, Clínica Universitaria Reina Fabiola, Córdoba, Argentina; 3 Instituto de Investigaciones en Ciencias de la Salud (INICSA), Consejo Nacional de Investigaciones Científicas y Técnicas (CONICET), Córdoba, Argentina; 4 Servicio de Endocrinología, Clínica Universitaria Reina Fabiola, Córdoba, Argentina; 5 Servicio de Neurocirugía, Clínica Universitaria Reina Fabiola, Córdoba, Argentina; 6 Department of Pediatrics, Washington University in St. Louis School of Medicine, Saint Louis, MO, United States

**Keywords:** collision tumor, pituicytoma, Cushing's disease, electron microscopy, case report

## Abstract

Collision tumors are rare conditions characterized by the coexistence of two histologically distinct neoplasms in the same region without histological admixture or an intermediate cell population zone. Our report represents one of the few studies on a collision tumor composed of an ACTH-secreting pituitary neuroendocrine tumor (PitNET) and a sellar pituicytoma. Here, we describe the clinical presentation, radiological findings, immunohistochemical/histopathological analysis, and ultrastructural examination of a 21-year-old woman with two different intracranial primary tumors at adjacent sites. Magnetic resonance imaging of the sellar region revealed a focal, 4 mm, nodular lesion in the left lateral recess that was hypointense on T2 and compatible with a micro-PitNET. Transnasal transsphenoidal resection was performed. Histopathological analysis revealed patterns consistent with a corticotroph PitNET, with diffuse cytoplasmic ACTH immunostaining and a Ki-67 index of 4%. Furthermore, the specimens included scattered adenohypophyseal and neural tissue. The latter was characterized by increased neoplastic proliferation consistent with a pituicytoma, confirmed by nuclear positivity for TTF-1 and a Ki-67 index of 3%. Ultrastructural analysis confirmed the coexistence of two morphologically distinct lesions. Postoperatively, the patient developed diabetes insipidus and remained in clinical and biochemical remission with no remaining tumor at nine months post-surgery. Pituitary collision tumors are sporadically reported and rare. Specifically, pituicytomas associated with pituitary hyperfunction are notably infrequent, and those linked to Cushing’s disease are extremely uncommon. This case highlights the exceptional rarity of sellar collision tumors and underscores the importance of reporting such cases to improve recognition, refine diagnostic strategies, and expand current understanding of complex pituitary tumor biology.

## 1 Introduction

A collision tumor is a rare neoplastic entity defined by the coexistence of two or more histologically distinct cell populations, either benign or malignant, arising within the same anatomical site while maintaining clearly identifiable histological, genetic, and biological characteristics without significant intermingling of tissues (1, 2).

Collision tumors involving pituitary neuroendocrine tumors (PitNETs) and other sellar neoplasms are exceptionally uncommon, and their clinicopathological features remain incompletely characterized (3). Preoperative identification of dual sellar pathology is particularly challenging, as most cases clinically and radiologically resemble conventional PitNETs (4). Clinically, presentation is primarily determined by the secretory activity of the adenohypophyseal component and, to a lesser extent, by the mass effect generated by the accompanying lesion. The marked overlap in clinical manifestations and imaging findings with typical PitNETs further limits preoperative recognition, and the definitive diagnosis is therefore usually established only through histopathological and immunohistochemical evaluation (5).

Pituicytomas are uncommon benign, solid, and well-circumscribed tumors found in the sellar and suprasellar regions, likely originating from pituicytes, which are unique glial cells of the neurohypophysis (6, 7). These tumors have been recognized as official WHO entities and are now included in both the WHO Classification of tumors of endocrine organs and the WHO Classification of tumors of the CNS (8, 9), where they are histologically classified as WHO Grade I tumors.

On the other hand, Cushing’s disease (CD) is a serious endocrine disease caused by excessive secretion of cortisol from the adrenal glands driven by excessive adrenocorticotropic hormone (ACTH) secretion from a corticotroph PitNET (10). The reported incidence ranges from 0.15 to 0.33 cases per million individuals per year (11) and is usually characterized by a substantial diagnostic delay, which contributes to the development of multiple and severe comorbidities, impaired quality of life, and increased mortality (12).

Reports of pituicytomas associated with pituitary hyperfunction in the literature are rare, and their association with CD is exceptionally uncommon. This study outlines the clinical presentation, radiological findings, immunohistochemical and histopathological analysis, and ultrastructural features of a cerebral collision tumor composed of two benign components located in the sellar region: an ACTH-secreting PitNET and a pituicytoma. This case was reported at a healthcare center in Córdoba, Argentina. A comprehensive medical history is crucial for determining the most appropriate treatment strategy for collision tumors.

## 2 Case description

A 21-year-old female patient was admitted to the Endocrinology Department of Clínica Universitaria Reina Fabiola due to central obesity, purple stripes, and increased facial and body hair. The patient denied having headaches or visual symptoms. She had no clinically relevant family or personal history, except for depression. On physical examination, body mass index was 28.5 kg/m² and blood pressure was 120/80 mmHg. She exhibited facial plethora, supraclavicular fullness, and hirsutism, without evident catabolic features suggestive of Cushing’s syndrome. Biochemical assessment demonstrated normal metabolism parameters: fasting plasma glucose 83 mg/dL (reference range [RR]: 70–110 mg/dL), serum creatinine 0.79 mg/dL (RR: 0.46–0.90 mg/dL), sodium 142 mEq/L (RR: 135–145 mEq/L), potassium 4.4 mEq/L (RR: 3.5–5.0 mEq/L), total calcium 10.3 mg/dL (RR: 9.0–10.7 mg/dL), and ionized calcium 1.16 mmol/L (RR: 1.16–1.32 mmol/L). Lipid profile revealed total cholesterol 189 mg/dL (RR: <200 mg/dL), HDL cholesterol 64 mg/dL (RR: >50 mg/dL), and LDL cholesterol 115 mg/dL (RR: <140 mg/dL). Serum 25-hydroxyvitamin D level was 27 ng/mL (RR: >30 ng/mL). Hormonal evaluation revealed elevated cortisol (30.2 μg/dL; RR: 3.7–19.4 μg/dL), ACTH (89.3 pg/mL; RR: 7.3–63.3 pg/mL), and total testosterone levels (0.71 ng/dL; RR: 0.12–0.48 ng/dL), whereas S-DHEA was within the control range (460 μg/dL; RR: 61.2–511.7 μg/dL). The urinary free cortisol concentration was 190.9 μg/day (RR: 4.3–176 μg/day). In addition, a loss of the circadian rhythm of cortisol was noted, confirmed by late-night salivary cortisol levels of 0.75 μg/dL (RR: <0.41 μg/dL). Moreover, the cortisol level did not decrease after a 1 mg dexamethasone suppression test (post-test cortisol: 39.7 μg/dL; RR: <1.8 μg/dL), strongly indicating CD (post-test cortisol: >5.0 μg/dL). An evaluation of other pituitary axes revealed isolated TSH suppression at 0.07 U/mL (0,27–4,2 U/mL) and low free T4 at 0.74 ng/dL (0,93–1,7 ng/dL), which was consistent with central hypothyroidism. Replacement therapy with 50 µg/day levothyroxine was initiated.

**Figure 1:**
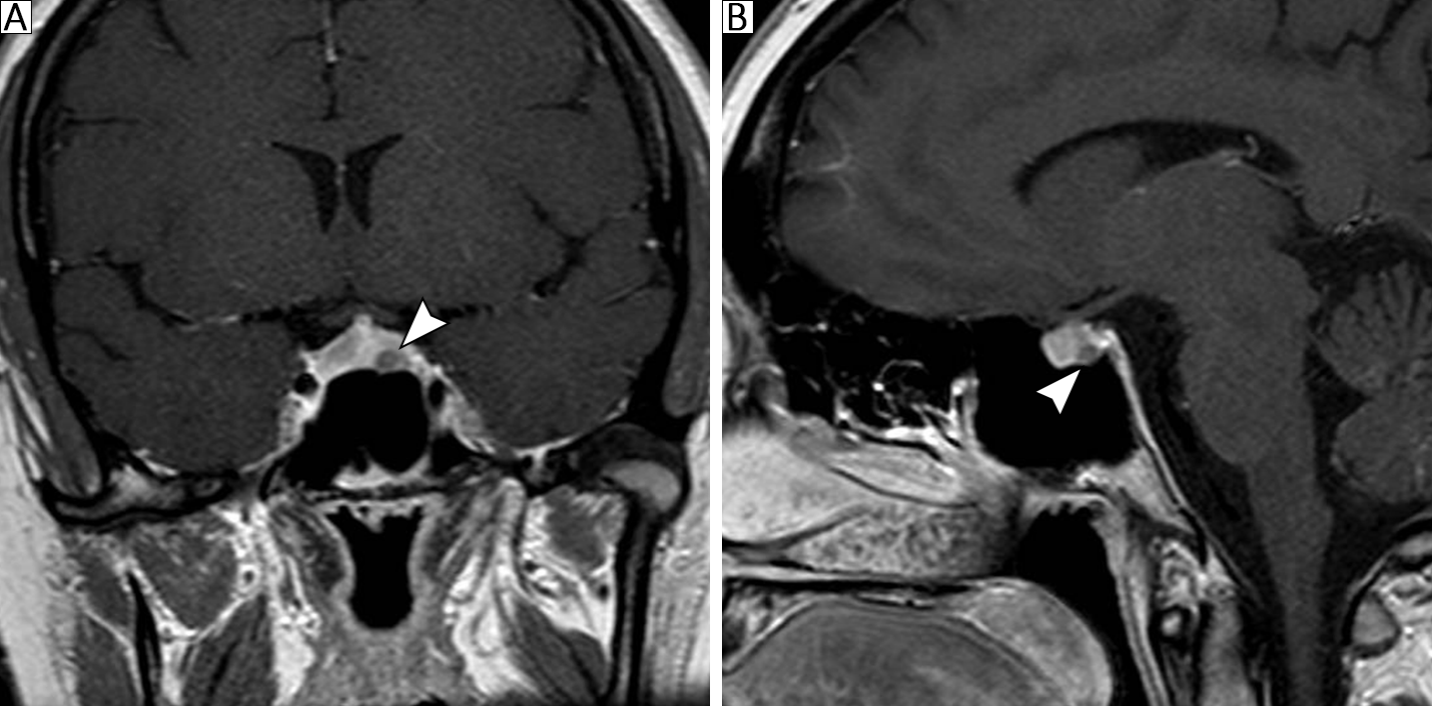
**(A, B)** Presurgical coronal and sagittal pituitary MR images showing a solid tumor lesion (4 mm) on the left side of the pituitary gland that was hypointense on T2-weighted images (white arrows). The lesion does not compress the pituitary stalk or the neurohypophysis.

Magnetic resonance imaging (MRI) of the sellar region revealed a globular appearance of the adenohypophysis, with a focal nodular image hypointense on T2-weighted sequences, located in the topography of the left lateral recess. The lesion exhibited well-defined borders, no enhancement on the post-gadolinium series, and a maximum diameter of 4 mm, which was consistent with a pituitary micro-PitNETs (**Figures 1A, B**). In addition, a hyperintense signal corresponding to the neurohypophysis was observed in its usual position.

**Figure 2:**
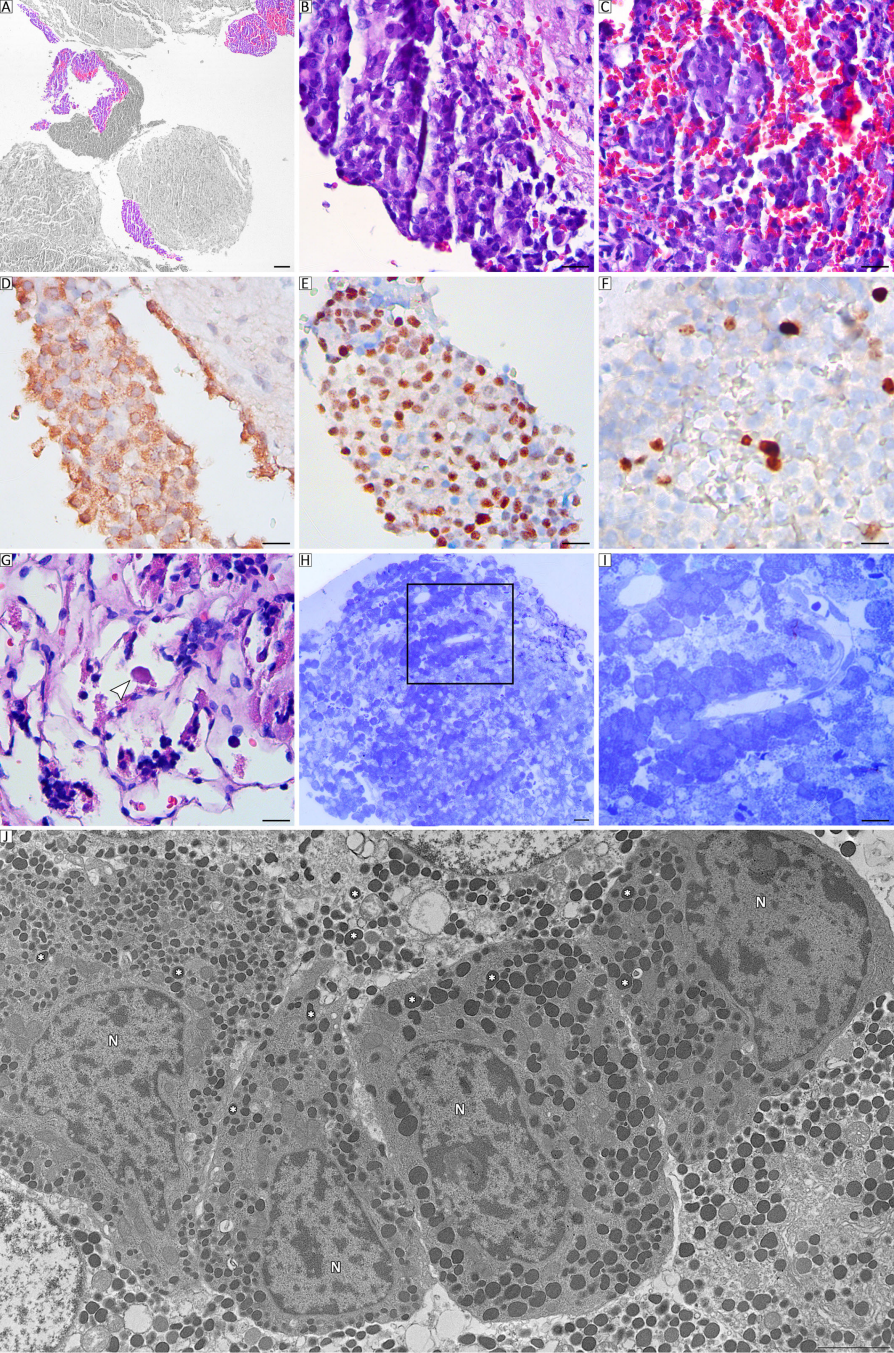
**(A)**. Panoramic histological image of a collision tumor detailing a corticotroph PitNET. (H/E staining). Scale bar, 100 μm. **(B, C)** Microscopic examination of PitNET showing neoplastic proliferation of epithelial basophilic corticotroph cells in a hemorrhagic background. (H/E staining). Scale bar, 20 μm. **(D)** Strong and diffuse immunolabeling for ACTH and T-Pit **(E)** was clearly detected in corticotroph PitNET cells (3, 3′-diaminobenzidine staining). Scale bar, 20 μm. **(F)** Nuclear immunohistochemical staining of Ki-67 proliferative activity in corticotroph PitNET (3, 3′-diaminobenzidine staining). Scale bar, 20 μm. **(G)** Details of nontumor basophilic corticotroph PitNET cells with degenerative changes: white arrow indicates typical Crooke-like changes (H/E staining). Scale bar, 20 μm. **(H, I)** Tumoral tissue is composed of clusters of medium-sized cells organized in a solid pattern around blood capillaries and containing numerous basophilic secretory granules (toluidine blue staining). Scale bar, 20 μm. **(J)** Ultrastructural analysis of densely granulated corticotroph PitNET cells reveals a remarkable accumulation of cytoplasmic secretory granules (asterisk) displaying variable morphology and electron density. N: nucleus. Scale bar, 2 μm.

**Figure 3:**
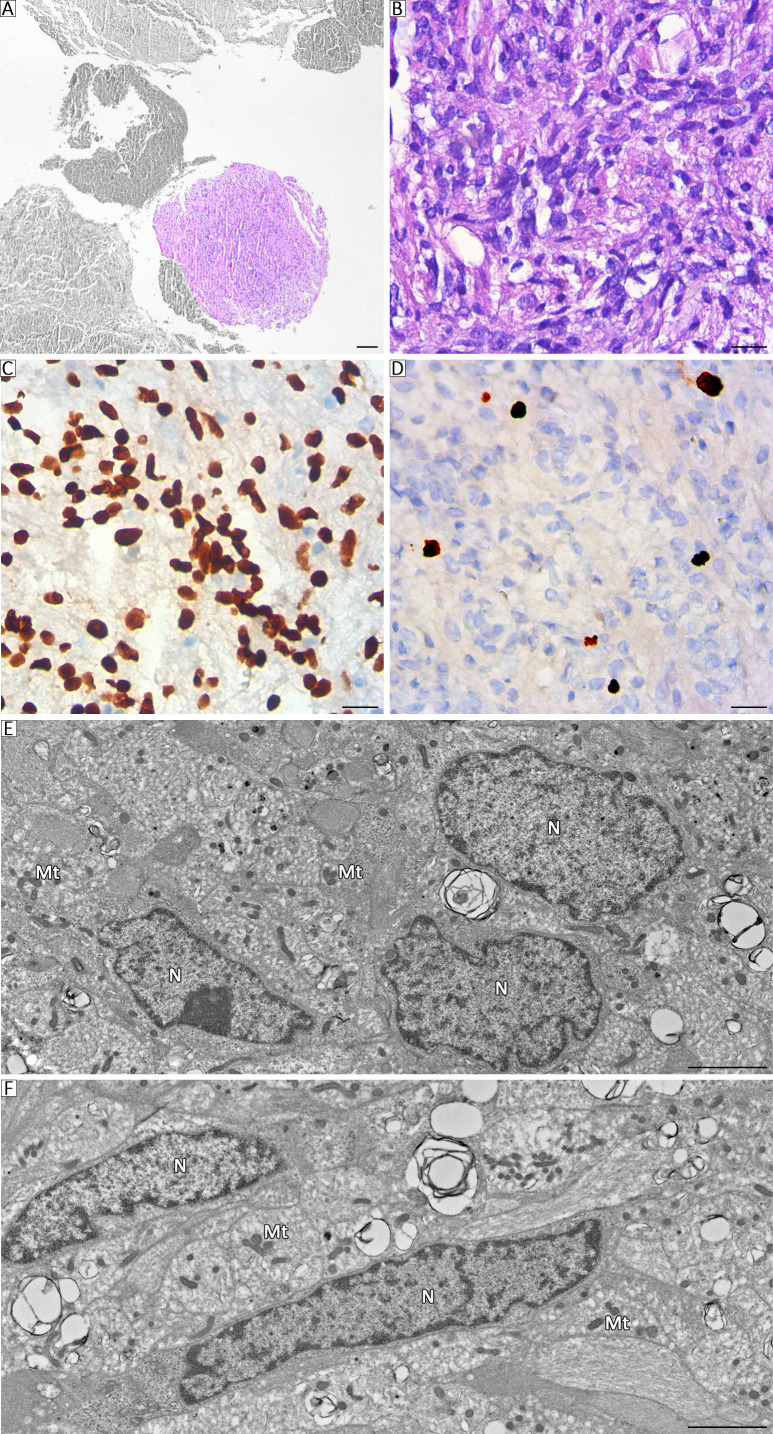
**(A)** Panoramic micrograph showing both neoplasms, with the pituicytoma highlighted. Scale bar, 100 μm. **(B)** Histopathological examination showing spindle cell neoplasm with a storiform pattern, short fascicles or spokes (H/E staining). Scale bar, 20 μm. **(C)** TTF-1 immunostaining depicting strong and diffuse nuclear positivity (3, 3′-diaminobenzidine staining). Scale bar, 20 μm. **(D)** Nuclear immunohistochemical staining of Ki-67 in neoplastic cells (3, 3′-diaminobenzidine staining). Scale bar, 20 μm. **(E, F)** Electron microscopy images of pituicytoma showing elongated cells with ovoid nuclei N, few organelles involved in protein synthesis, and mitochondria (Mt), along with an absence of secretory granules. Scale bar, 20 μm.

The patient underwent surgical resection via an endoscopic transsphenoidal approach. Histopathological analysis revealed two independent adjacent proliferative processes. One of them exhibited epithelial neoplastic proliferation characterized by cuboidal to polygonal medium-sized cells with monomorphic morphology, which were arranged in a solid pattern interspersed with delicate, well-vascularized connective tissue septa (**Figures 2A–C**). Immunohistochemically, ACTH was diffusely positive with intense granular cytoplasmic staining (**Figure 2D**), T-Pit was diffusely positive with nuclear staining (**Figure 2E**), and Ki-67 was positive in 4%, together corresponding to a corticotroph PitNET (**Figure 2F**). Furthermore, the material included scarce preserved adenohypophyseal tissue, revealing basophilic corticotroph cells with changes in Crooke’s hyaline (**Figure 2G**). Similarly, high-resolution optical microscopy of semithin (200 nm) sections from Araldite-embedded pituitary glands revealed epithelial cells with eccentric nuclei and finely granular cytoplasm, exhibiting a homogeneous pattern with toluidine blue staining. These findings are consistent with the storage of ACTH-secretory granules (**Figures 2H, I**). By transmission electron microscopy, corticotroph tumoral cells with ovoid or irregular nuclei were observed. The cell cytoplasm also exhibited prominent accumulation of secretory granules (150–450 nm in diameter), with marked variability in electron density and a teardrop shape in some of them. Their abundance is consistent with the diagnosis of a densely granulated corticotroph PitNET (**Figure 2J**). The other process, ~2 mm in diameter, involved astroglial cellular proliferation, with elongated elements arranged in a short interwoven fascicular pattern, occasionally forming whorls within a loose fibrillar stroma (**Figures 3A, B**). Immunohistochemically, ACTH was negative, thyroid transcription factor 1 (TTF-1) was diffusely positive with nuclear staining, and the Ki-67 index was 3%, which is consistent with a classic pituicytoma (**Figures 3C, D**). Electron microscopy revealed elongated cells with indented euchromatic nuclei and prominent nucleoli. Phagolysosomes containing electron-dense material and membranous debris, a small number of elongated mitochondria, and free ribosomes that accumulated in the cytoplasm were also observed. In addition, the accumulation of intermediate filaments was observed in some cells (**Figures 3E, F**).

During the immediate postoperative period, the patient had a cortisol level of 2.02 µg/dL and an ACTH level of 19.5 pg/mL, and a 30 mg/day hydrocortisone replacement therapy was initiated. She also developed diabetes insipidus, which required transient desmopressin treatment. Eleven months after surgery, the patient remains in clinical and biochemical remission of the disease, with neuroimaging showing no residual tumor.

## 3 Discussion

In the sellar region, PitNETs are the most common lesions derived from the pituitary gland. Additionally, spindle cell tumors of the posterior pituitary (pituicytoma, spindle cell oncocytoma, and granular cell tumor) can also be found in this region (9). In this context, collision sellar lesions refer to cases where a PitNET coexists with another pituitary or sellar lesion (13). This well-defined entity, described in the *sella turcica* region, involves multiple associations but rarely the concurrent appearance of a PitNET and a pituicytoma.

In particular, the occurrence of pituicytomas associated with pituitary hyperfunction is significantly limited, and those associated with CD are extremely rare. A recent review reported 13 pituicytoma cases with PitNET confirmed in only 5 of them (14). The present case represents a rare example of a collision tumor involving an ACTH-secreting PitNET and a pituicytoma, combining adenohypophyseal and neurohypophyseal neoplastic components within the sellar region. Collision tumors of this type are exceptionally uncommon, and their recognition relies primarily on careful histopathological examination. In our study, a 21-year-old female patient with CD underwent surgical resection of two independent adjacent sellar proliferative processes. Histopathologically, a pituicytoma and a concurrent corticotroph PitNET were diagnosed. The mechanism behind the development of pituitary collision tumors is still unclear. One hypothesis suggests that both tumor types share common factors contributing to tumorigenesis (15). The coexistence of these cells could indicate common progenitor cells, possibly stellate follicular cells, which are thought to regulate the activity of anterior pituitary endocrine cells through the production of cytokines and growth factors (16, 17). Stellate follicular cells also express BCL-2, an oncoprotein that inhibits apoptosis and plays a role in the progression of various tumors (18). Some cases of pituicytomas have shown focal expression of BCL-2 (19). In contrast, mature follicule-stellate cells are known not to express TTF-1 (20). Another possible explanation is the production of stimulating signals (cytokines and/or growth factors) by pituicytoma cells, which, by acting on adjacent neurosecretory adenohypophyseal cells, may facilitate the development of hypersecretion syndromes (19, 21).

Pituicytomas account for approximately 0.07% of all primary tumors in the sellar region (22). They predominantly occur in adults, typically between the fifth and sixth decades of life, with a slight male predominance (23). In a minority of cases, pituicytomas are discovered incidentally (24). Despite the radiological overlap, pituicytomas typically exhibit a suprasellar or sellar epicenter and appear as well-circumscribed solid lesions that are isointense on T1-weighted images and hyperintense on T2-weighted sequences, with intense and homogeneous gadolinium enhancement reflecting their marked vascularity (25–27). In contrast, corticotroph micro-PitNETs are usually small intrasellar lesions that demonstrate relative hypointensity on dynamic contrast-enhanced imaging compared with the normal pituitary gland (28). Since it does not present specific clinical, radiological, or macroscopic characteristics, histopathological analysis is essential to differentiate this lesion from another tumor, including a clinically non-functioning PitNET. In our patient, the pituicytoma was identified as an incidental finding during surgery for a functional corticotroph PitNET. The extremely small size of the lesion suggests an early-stage neoplasm and illustrates how neurohypophyseal tumors may remain clinically silent, becoming detectable only in the context of surgery for hormonally active pituitary lesions.

Different tumor subtypes are distinguished: pituicytoma, granular cell tumors of the sellar region, spindle cell oncocytoma, and ependymal pituicytoma, all of which are part of a spectrum of tumors whose methylation profile and nuclear expression of TTF1 are similar (29). The histology of our case aligns with that of classic pituicytoma, showing elongated bipolar cells in short intertwined fascicles with a fibrillar background but lacking the Rosenthal fibers and eosinophilic granular bodies observed in pilocytic astrocytoma. In contrast, granular cell tumors consist of polygonal cells with acidophilic cytoplasm and PAS-positive granules, forming solid layers or irregular fascicles with perivascular lymphocytic infiltrates. Spindle cell oncocytoma features fusiform or epithelioid cells in an intertwined fascicular pattern with intensely acidophilic cytoplasm and variable granularity (30). The immunohistochemical profile revealed positive expression of TTF1, vimentin, S100, and focal GFAP in all patients, which was associated with a low proliferation index (Ki-67 < 2–3%). Variable marking for EMA, CD68, and BCL-2, among others, is also recognized (20). Thus, the diagnosis and treatment of pituitary collision tumors are often challenging and limited to surgery and histopathological examination. For the case reported here, the integration of morphological and immunohistochemical findings was essential for diagnosis. Nuclear TTF-1 expression confirmed neurohypophyseal lineage, and the presence of a detectable Ki-67 proliferative index supported the neoplastic nature of the pituicyte proliferation. Although the immunohistochemical panel was limited due to the extremely small amount of available tissue, the combined histological and immunophenotypic features were sufficient to establish the diagnosis of fibrillary pituicytoma. Also, transmission electron microscopy offered invaluable additional insights enabling a more precise and detailed characterization at the ultrastructural level. The pituicytoma ultrastructure shares characteristics with that of its normal counterpart, that is, elongated or oval cells with focal cytoplasmic accumulation of intermediate filaments without interdigitated membranes or secretory granules. The oncocytic variant also has abundant mitochondria (17, 20). Despite being previously described, Cenacchi and colleagues (16) reported the morphological characteristics of endocrine differentiation (secretory granules) in a pituicytoma (16), which could explain the endocrine symptoms in cases of hormonal hypersecretion and pituicytoma. From a surgical perspective, while gross total resection is the primary objective in pituicytomas (often limited by their vascularity and close relationship with the pituitary stalk) (31, 32) the main goal in corticotroph PitNETs is biochemical remission, with selective adenoma resection generally being more predictable, particularly in well-identified microadenomas (33, 34).

Diagnosing CD is particularly challenging because of the variability in its clinical presentation and, more importantly, the lack of specific symptoms and signs to differentiate it in patients. In our case, the patient presented with CD with biochemical confirmation, a histological study confirming a corticotropic PitNET, and an incipient 2 mm pituicytoma with a Ki-67 index of 3%. In line with our findings, the presence of pituicytomas associated with CD corresponds to lesions of <10 mm and a Ki-67 index of 1–2% (35).

In addition, electron microscopy is routinely used to assess granularity patterns, facilitating the differentiation between densely granulated (DG) and sparsely granulated (SG) tumors. A recent cohort study confirmed that DG corticotroph tumors are more prevalent than SG tumors and are predominantly micro-PitNET (36), which aligns with our findings. Moreover, DG tumors have been reported to be more frequently symptomatic and associated with higher remission rates, as observed in our case (37, 38). In line, transmission electron microscopy provided key confirmation of the DG corticotroph PitNET by demonstrating abundant cytoplasmic secretory granules, complementing histological and immunohistochemical findings and supporting precise WHO subtyping of the PitNET.

Regular follow-up with MRI is recommended for patients with residual tumors because of their slow growth and low proliferative activity. As with PitNET, regular endocrinological follow-up is needed for patients with pituicytomas to assess potential hypothalamo-pituitary insufficiency.

In summary, we described a very rare collision sellar tumor: ACTH-secreting PitNET and pituicytoma. This case highlights that even very small neurohypophyseal proliferations displaying increased cellularity, characteristic spindle-cell architecture, and TTF-1 expression should be carefully evaluated, as these integrated features may reveal an incipient pituicytoma within a collision tumor rather than normal posterior pituitary tissue. This coexistence appears to be more frequent than expected, suggesting the involvement of shared endocrinological mechanisms that are not yet fully understood. Recognizing this association is essential for optimizing differential diagnosis, particularly in patients with CD, where the simultaneous occurrence of both lesions can complicate clinical and radiological assessment. We emphasize the crucial role of electron microscopy, which offers additional evidence for confirming the pathological diagnosis and ensuring greater therapeutic accuracy in the management of patients. Additionally, long-term postoperative monitoring is crucial due to the potential for recurrence and the risk of hypopituitarism. We are confident that a better understanding of the molecular and genetic pathogenesis of pituicytomas will allow for the design of more specific and effective personalized treatments in the short to medium term.

## 4 Conclusions

The comprehensive analysis of this clinical case highlights that collision tumors of the sellar region, although rare, represent a significant diagnostic challenge, as their clinical and radiological features frequently mimic those of a conventional PitNET. The coexistence of an ACTH-secreting PitNET and a pituicytoma constitutes an exceptionally uncommon entity, whose preoperative identification proved nearly impossible, underscoring that definitive diagnosis relies strictly on detailed histopathological and immunohistochemical evaluation. From a pathogenetic perspective, the presence of two independent yet adjacent neoplasms suggest complex biological mechanisms that remain incompletely understood. Ultimately, reporting such exceptional cases is crucial for expanding medical knowledge and may contribute to the development of personalized therapeutic strategies based on the molecular and genetic pathogenesis of these tumors in the near future.

## Data Availability

The raw data supporting the conclusions of this article will be made available by the authors, without undue reservation.
